# Metabarcoding reveals a hidden endophytic stage of the 'zombie-ant' fungus in Amazonian mosses

**DOI:** 10.3897/imafungus.17.196998

**Published:** 2026-07-14

**Authors:** Tales Alves-Júnior, João Paulo Machado de Araújo, Aristóteles Góes-Neto, Thairine Mendes-Pereira, Charles E. Zartman

**Affiliations:** 1 Programa de Pós-Graduação em Botânica, Instituto Nacional de Pesquisas da Amazônia, Manaus, Brazil Departamento de Microbiologia, Instituto de Ciências Biológicas, Universidade Federal de Minas Gerais Belo Horizonte Brazil; 2 Natural History Museum Denmark, University of Copenhagen, Copenhagen, Denmark Programa de Pós-Graduação em Botânica, Instituto Nacional de Pesquisas da Amazônia Manaus Brazil; 3 Departamento de Microbiologia, Instituto de Ciências Biológicas, Universidade Federal de Minas Gerais, Belo Horizonte, Brazil Natural History Museum Denmark, University of Copenhagen Copenhagen Denmark; 4 Departamento de Entomologia, Universidade Federal de Viçosa, Viçosa, Brazil Departamento de Entomologia, Universidade Federal de Viçosa Viçosa Brazil

**Keywords:** amplicon sequencing, co-evolution, death-grip, mycobiome, niche selection

## Abstract

Hypocrealean fungi include numerous entomopathogens, notably the hyperdiverse genus *Ophiocordyceps*, in which lineages manipulate insect hosts to die in specific microhabitats, such as mosses, which may facilitate spore dispersal. A dual endophyte-entomopathogen lifestyle has been proposed for *Ophiocordyceps* from temperate and subtropical systems; however, megadiverse tropical ecosystems remain poorly explored. Our study aims to characterize fungal communities in central Amazonian bryophytes from the Adolpho Ducke Forest Reserve in order to investigate the alleged dual lifestyle within a hyperdiverse tropical environment. Four sample types were analyzed: 1) *Ophiocordyceps* from the host insect; 2) bryophyte leaves at the host insect biting site; 3) bryophyte leaves adjacent to (≥ 5 cm) the biting site; 4) and bryophyte leaves (≥ 10 m) from the same locality within the study site. Total genomic DNA was extracted, the TEF1-α region was amplified, and amplicons were sequenced using Oxford Nanopore technology. Metabarcoding of 45 libraries revealed structured fungal communities within Amazonian bryophytes, resulting in the identification of 2,277 fungal taxa. Across all bryophyte samples, we report a significant detection of entomopathogenic fungi, mostly comprising ant-specific *Ophiocordyceps* (e.g., *O. camponoti-nidulantis* and *O. kniphofioides*). Together, these results support the hypothesis of a ubiquitous endophytic stage of myrmecophilous *Ophiocordyceps* among Amazonian bryophytes, indicating a closer link between host manipulation and plant association than previously recognized.

## Introduction

Hypocrealean fungi (*Ascomycota*, *Hypocreales*) are notable for their ecological plasticity and remarkable diversity of life strategies, ranging from saprophytism and parasitism to mutualistic nutritional symbiosis among arthropods and plants (Araújo and Hughes 2016; Matsuura et al. 2018; Wang et al. 2020). Invertebrate-infecting lineages are termed entomopathogenic fungi (EPF) and belong primarily to four families in *Hypocreales*: *Clavicipitaceae*, *Cordycipitaceae*, *Polycephalomycetaceae* and *Ophiocordycipitaceae* (Sung et al. 2007a, 2007b; Xiao et al. 2023).

*Ophiocordyceps* Petch (*Ophiocordycipitaceae*) is a hyperdiverse genus of entomopathogenic fungi, presently comprised of more than 350 described species (mycobank.org); however the full extent of its diversity remains unknown. The genus was originally circumscribed by Petch (1931), and throughout its evolutionary history, *Ophiocordyceps* evolved to infect and kill arthropods among at least 13 orders of insects (Araújo and Hughes 2016; Araújo et al. 2022). Specifically, *Hymenoptera*-infecting lineages, such as those associate with *O. unilateralis* s.l. and the *Neocordyceps* clade, also evolved this remarkable ability to manipulate host behavior (Evans et al. 2011; Araújo et al. 2018, 2020).

Behavioral manipulation is recognized as an *extended phenotype*, a concept originally coined by Dawkins (1982) in which parasitoid genes manifest into the host’s phenotype, aiming to benefit the parasitoid in its development, reproduction, and transmission (Hughes et al. 2011). For example, the species belonging to the *O. unilateralis* clade, after infecting its ant host, colonizes its entire body while also downregulating the host’s immune system. thus producing an array of neuromodulators, psychoactive alkaloids, and tremorgenic terpenoids that subsequently disrupt the host’s physiological and social functions (de Bekker et al. 2014a, 2014b, 2015; Hughes et al. 2016; Fredericksen et al. 2017). These compounds induce the host to “self-sabotage” with behaviors which include abandoning its colony with the ultimate goal of “seeking” microclimates that are optimal for fungal growth and dispersal. This final event culminates in a behavioral phenomenon known as the ‘death-grip’ wherein the insect bites onto a substrate before sporulation (Hughes et al. 2011; de Bekker et al. 2015; Araújo and Hughes 2019; Andriolli et al. 2019; Somavilla et al. 2019, 2020).

Death site selection by infected hosts is influenced by abiotic factors such as temperature, relative humidity, canopy openness, and height, all of which vary along a latitudinal gradient (Andersen et al. 2009; Loreto et al. 2018; Andriolli et al. 2019; Cardoso Neto et al. 2019). Nevertheless, infected ants exhibit distinct preferences for specific substrates as their death sites, clinging to structures ranging from twigs to leaves, thorns, and bryophytes, depending on the insect species (Araújo et al. 2018). In the Amazon, ant-infecting species such as *O. camponoti-renggeri*, *O. monacidis*, *O. kniphofioides* s.s., and *O. odontomachi* (and their respective hosts) display pronounced fidelity to bryophytes as their final attachment sites, suggesting a coevolutionary adaptation linking fungal dispersal strategies to specific microhabitats (Araújo et al. 2015, 2018, 2020; Sanjuan et al. 2015).

Bryophytes (*Bryophyta* sensu lato), as well as vascular plants, harbor in their phyllosphere entire fungal communities of both *Ascomycota* (e.g., *Octospora*, *Lamprospora*, *Epibryon*) and *Basidiomycota* (e.g., *Galerina*, *Rickenella*, *Mycena*), adopting roles as biotrophs, necrotrophs or endophytes (Döbbeler 1978; Felix 1988; Davey and Currah 2006; Davey et al. 2013a, 2013b; Döbbeler and Hertel 2013; Zhang et al. 2018). These ‘bryophilous fungi’ often exhibit ecological versatility, with some lineages displaying more than one lifestyle. Metagenomic studies in temperate regions (e.g., Canada, Norway) reveal entomopathogenic genera such as *Ophiocordyceps* and related *Ophiocordycipitaceae* found within bryophytes (Davey et al. 2014; Matsuoka et al. 2021).

Even predominantly entomopathogenic species such as the ‘caterpillar fungus’ (*O. sinensis*) exhibit unexpected plasticity, as illustrated by it being found within *Syntrichia*, a moss that was also observed to be consumed by the caterpillar host (Wang et al. 2020), consistent with recent proposals that *Ophiocordyceps* may adopt facultative endophytic phases as part of its reproductive strategy (Wang et al. 2022; de Menezes et al. 2023; Chen et al. 2024). Adding complexity to this picture, several *Ophiocordyceps* species display heterothallism, requiring compatible mating-type loci for sexual reproduction (Zou et al. 2019; Xu et al. 2023; Li et al. 2024a, 2025; Zou et al. 2024), which may impose additional selective pressure on host and habitat range. However, these insights derive almost exclusively from temperate and subtropical ecosystems, leaving key assumptions untested in hyperdiverse tropical regions such as the Amazon, where high humidity, host diversity, and bryophyte richness could drive unexplored fungal adaptations and lifestyles.

Recent field observations describe ant-infecting lineages of *Ophiocordyceps* that appear to target specific plants, such as *Tillandsia* bromeliads (Will et al. 2023a), or even understory palm trees (Andriolli et al. 2025) as their death site. Araújo et al. (2018, 2020) observed a consistent preference particularly for *Octoblepharum* in Amazonia as the death sites for *Ophiocordyceps*-infected ants. Unusually, *O. monacidis*, one of the species commonly found buried in *Octoblepharum* moss carpets, also had ascomata closely resembling the morphology of sporophytes of the genus (Araújo et al. 2018).

In light of these findings, our study aims to address the following questions: (1) How are endophytic fungal communities in Amazonian mosses (*Bryophyta* sensu lato) structured? (2) Do endophytic fungal communities in Amazonian mosses contain common entomopathogenic fungi whose insect hosts are consistently manipulated to perform a death grip on moss leaves? In the interest of investigating such questions, we characterized the fungal communities associated with bryophytes in a forest reserve in the Central Amazon through metabarcoding and screening for entomopathogenic fungi such as *Ophiocordyceps*, in order to refine our understanding of the factors contributing to host substrate selection and ecological associations within this fungal genus.

## Methods

### Study area

**Figure 1.**
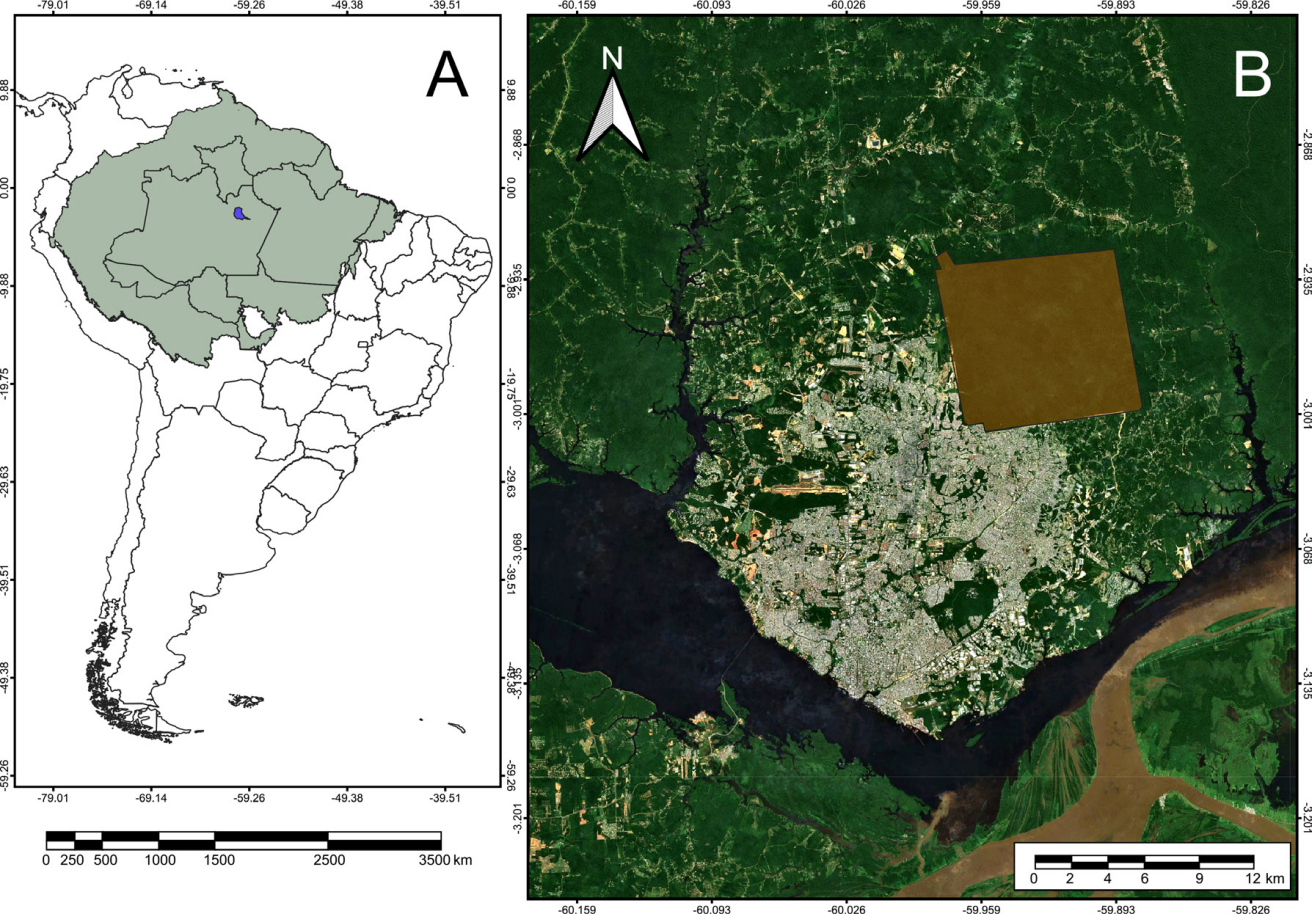
Map of the study area, located north of Manaus, Amazonas, Brazil. **A** Study area location within the Amazon biome (in Green) in South America, and the city of Manaus (in Blue); **B** Satellite imagery (Sentinel-2) of the Adolpho Ducke Forest Reserve (in Orange) within Manaus. Tick marks at the map frame represent SIRGAS 2000 / UTM Zone 21S projected coordinates.

This study was undertaken at the Adolpho Ducke Forest Reserve, located on the north side of the city of Manaus, state of Amazonas, Brazil (Fig. 1). The Reserve has an area of ca. 10,000 ha, consisting mainly of vegetation such as upland (*Terra-Firme*), white-sands (*Campinarana*) and secondary (*Capoeira*) forest types. The climate of the area is classified as tropical wet climate (Köppen-Geiger: Af), having its rainy season between December and March, and dry season between June to November, with an average annual precipitation of 2300 mm, average temperature of 26 °C and relative humidity of 84% (Marques Filho et al. 1981; Beck et al. 2018).

### Field sampling

**Figure 2.**
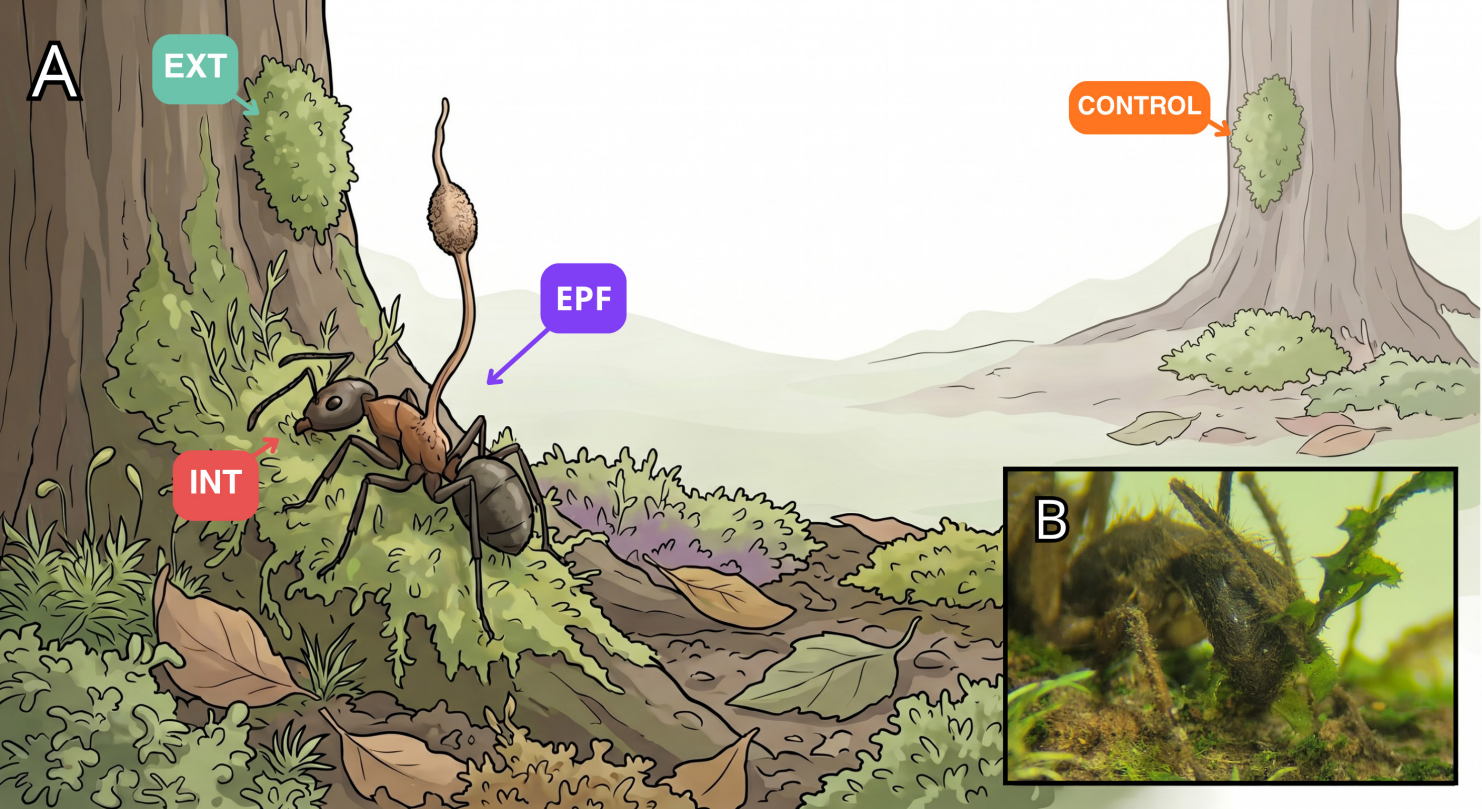
Graphic schematic of the experimental sampling design. **A** Illustrative overview of the sampling layout within a tropical forest understory. Color-coded arrows indicate sampling categories: INT (bitten bryophyte), EXT (adjacent bryophyte, ≥ 5 cm), EPF (entomopathogenic fungus), and CONTROL (bryophyte ≥ 10 m from any infected cadaver). Illustration produced with the assistance of GenAI (Gemini Pro 3.1, Google); **B** Macro-photograph of an *Ophiocordyceps*-infected ant cadaver (EPF) biting onto a bryophyte (INT).

The experimental sampling protocol consisted of surveying in “ant graveyards” (i.e. areas containing high density of *Ophiocordyceps*-infected specimens, epizootics), with the purpose of carefully inspecting the understory (0–2 m) for infected insects attached to bryophytes as their biting location prior to death. Subsequently, samples were split into four categories (Fig. 2): (EPF = Entomopathogenic Fungus) *Ophiocordyceps* emerging from the insect, (INT) bryophytes at the biting site, (EXT) bryophytes adjacent (≥ 5 cm) to the biting site, and (CONTROL) bryophytes collected at ≥ 10 meters distance from the biting site (control) that did not exhibit any association with *Ophiocordyceps*-infected insect cadavers (see Table 1).

**Table 1.** Specimens found attached to bryophytes during the field expeditions. Exemplars highlighted in bold were successfully sequenced. HA = Harpy Eagle Trail, Section “A”. HB = Harpy Eagle Trail, Section “B”. AC = Acará Trail. B = “Baixio/Areal” Trail. MET = Meteorology station trail.

**ID**	**Species**	**Host**	**Moss Substrates**	**Locality**
*TAJ30*	*Ophiocordyceps curculionum*	*Piazurus melanostictus* (*Coleoptera*, *Curculionidae*)	*Octoblepharum* sp.	HA
*TAJ31*	*Ophiocordyceps kniphofioides* s.l.	*Lampyridae* sp. larvae (*Coleoptera*)	*Octoblepharum* sp.	B
*TAJ32*	*Ophiocordyceps camponoti-renggeri*	*Camponotus renggeri* (*Hymenoptera*, *Formicidae*)	*Plagiochila* sp.; *Octoblepharum* sp.	HA
*TAJ33*	*Ophiocordyceps* sp.	*Pintalia* sp. (*Hemiptera*, *Cixiidae*)	*Lejeuneaceae* sp.	B
***TAJ59***	***Ophiocordyceps camponoti-nidulantis***	***Camponotus nidulans* (*Hymenoptera*, *Formicidae*)**	***Octoblepharum* sp**.	**AC**
***TAJ60***	***Ophiocordyceps camponoti-nidulantis***	***Camponotus nidulans* (*Hymenoptera*, *Formicidae*)**	***Lejeuneaceae* sp**.	**AC**
***TAJ61***	***Ophiocordyceps cf. camponoti-balzani***	***Camponotus* cf. *balzani* (*Hymenoptera*, *Formicidae*)**	***Octoblepharum* sp**.	**AC**
***TAJ63***	***Ophiocordyceps camponoti-nidulantis***	***Camponotus nidulans* (*Hymenoptera*, *Formicidae*)**	***Octoblepharum* sp**.	**AC**
*TAJ64*	*Ophiocordyceps kniphofioides*	*Cephalotes atratus* (*Hymenoptera*, *Formicidae*)	*Octoblepharum* sp.	AC
***TAJ65***	***Ophiocordyceps cf. camponoti-renggeri***	***Camponotus cf. renggeri* (*Hymenoptera*, *Formicidae*)**	***Octoblepharum* sp**.	**AC**
*TAJ69*	*Ophiocordyceps camponoti-renggeri*	*Camponotus renggeri* (*Hymenoptera*, *Formicidae*)	*Octoblepharum* sp.	HB
***TAJ70***	***Ophiocordyceps cf. camponoti-chartificis***	***Camponotus chartifex* (*Hymenoptera*, *Formicidae*)**	***Lejeuneaceae* sp**.	**HA**
***TAJ71***	***Ophiocordyceps* sp1**.	***Neoponera cf. cavinodis* (*Hymenoptera*, *Ponerinae*)**	***Lejeuneaceae* sp**.	**HB**
***TAJ72***	***Ophiocordyceps australis***	***Neoponera cf. cavinodis* (*Hymenoptera*, *Ponerinae*)**	***Octoblepharum* sp**.	**HB**
***TAJ74***	***Ophiocordyceps* sp1**.	***Neoponera cf. cavinodis* (*Hymenoptera*, *Ponerinae*)**	***Lejeuneaceae* sp**.	**B**
***TAJ75***	***Ophiocordyceps* sp1**.	***Neoponera cf. cavinodis* (*Hymenoptera*, *Ponerinae*)**	***Leucomium* sp**.	**B**
***TAJ76***	***Ophiocordyceps kniphofioides***	***Cephalotes atratus* (*Hymenoptera*, *Formicidae*)**	***Octoblepharum* sp**.	**B**
*TAJ101*	*Ophiocordyceps evansii*	*Neoponera foetida* complex (cf. *curvinodis*) (*Hymenoptera*, *Ponerinae*)	*Ceratolejeunea* sp.	MET
*TAJ102*	*Ophiocordyceps evansii*	*Neoponera foetida* complex (cf. *curvinodis*) (*Hymenoptera*, *Ponerinae*)	*Ceratolejeunea* sp.	MET

Samples were subsequently photographed individually, using either a Nikon D90 fitted with an AF Micro NIKKOR 60 mm f/2.8D Lens and a Godox MF-R76 macro ring Flash, a Galaxy S20FE Smartphone Camera or a Leica EZ4 stereomicroscope so that the fungi, host and respective focal bryophyte could be identified later by morphology and host associations (fungi-insect).

Subsequently, the specimens were cleaned of any coarse debris, and placed in 2 mL microcentrifuge tubes, and stored in a freezer. In compliance with Brazilian legislation regarding biodiversity sampling and transportation, permits were provided by the Instituto Chico Mendes de Conservação da Biodiversidade through the Biodiversity Authorization and Information System (SISBIO/ICMBio, Authorizations 87724–1 and 87721–2).

### DNA extraction and PCR amplification

The samples (EPF, INT, EXT and CONTROL) were surface sterilized using 70% ethanol for 1 min, followed by 5% sodium hypochlorite for 1 min and sterile distilled water for 1 min (Lacey 2012). Each sample was then dried on sterile filter paper, placed in a 2 mL microtube and macerated in contact with liquid nitrogen. Afterwards, samples were transferred to tubes containing a lysin matrix from ZymoBIOMICS^TM^ DNA Miniprep Kit (Zymo Research, California, USA), and total genomic DNA (gDNA) was extracted using the manufacturer’s instructions. The quality of the resulting gDNA was evaluated through 1% agarose gel electrophoresis at 100 V/400 mA for 30 min, with a quantification assay performed using Invitrogen Qubit™ 1X dsDNA Broad Assay kit on the Qubit 4 Fluorometer (ThermoFisher Scientific, Massachusetts, USA), to make sure adequate concentrations of gDNA were available (1 ng per sample) (Fonseca et al. 2022).

Although ITS is widely used as the primary fungal barcode, it can provide limited discriminatory power for some groups, in which secondary loci are usually recommended where this marker does not provide adequate resolution (Lücking et al. 2020). Additionally, for several *Ophiocordyceps* species, ITS is known to yield intragenomic variants (paralogs) and non-functional copies (pseudogenes) of itself (Li et al. 2017), those which in a metabarcoding context can, for instance, be amplified and resolved as distinct sequences, artificially inflating diversity and affecting species-level assignment, inferences which our work relies on. ITS is also one of the least-represented markers for *Ophiocordyceps* in public repositories, so reference coverage for the genus is sparse even where the locus might otherwise discriminate.

We therefore targeted the TEF1-α region as our barcode. A highly conserved, single-copy protein-coding gene, TEF1-α provides a stronger phylogenetic signal and higher species-level resolution than ITS while avoiding the pseudogene-driven diversity inflation noted above. It was formally adopted as the secondary fungal barcode precisely to address a key limitation of ITS, which accurately identifies only about three-quarters of fungal species and often cannot separate closely related taxa (Meyer et al. 2019). TEF1-α also reliably resolves closely related species within *Hypocreales* (Yörük and Yli-Mattila 2024), serves as a core systematic marker for *Cordyceps*, *Ophiocordyceps* and related hypocrealean entomopathogens (Sung et al. 2007a, 2007b), and is a stable, conserved single-copy locus in *Ophiocordyceps* itself (Tong et al. 2023). It also offers a key practical advantage for our target: among the sequences available for *Ophiocordyceps* in public repositories, it is one of the best represented, giving comparatively broad reference coverage for this genus.

The extracted gDNA from each subunit was then amplified using the TEF1-α primers 983F (5’ GCYCCYGGHCAYCGTGAYTTYAT 3’) and 2218R (5’ ATGACACCRACRGCRACRGTYTG 3’) (Rehner and Buckley 2005). Amplification was performed using a Veriti™ Thermal Cycler (ThermoFisher Scientific) in a 50 µL PCR Reaction that contained 25 µL of Kapa Taq ReadyMix 2x (KK1006), 1.5 µL of the forward and reverse primers at 10 µM, 18 µL of ultrapure water and 4 µL of the gDNA. To amplify our target region, PCR conditions were as follows: 2 min at 94 °C, 10 cycles of 94 °C for 30 s, 64 °C for 1 min, and 72 °C for 1 min; followed by 35 cycles of 94 °C for 30 s, 54 °C for 1 min, and 72 °C for 1 min; final extension of 3 min at 72 °C (Araújo et al. 2018). Amplification of TEF1-α region was checked through 1% conventional agarose gel electrophoresis at 100 V/400 mA for 25 min.

### PCR cleaning, library preparation and metagenomic sequencing

Resulting PCR products were purified using AMPureXP magnetic beads (Beckman Coulter Inc., USA) to remove short fragments, unincorporated dNTPs and other contaminants. Sequencing libraries were then prepared using the Oxford Nanopore Technologies (ONT, UK) Ligation Sequencing Kit SQK-LSK109, following the manufacturer’s protocol. To enable multiplex sequencing, samples were barcoded with ONT’s Native Barcoding Kits (EXP-NBD104 and EXP-NBD114), ensuring precise sample identification during downstream analysis. We then quantified the final DNA content of the finished libraries using a Qubit 4 Fluorometer. The concentration of each library was adjusted before sample ligation to each barcode to ensure the same amount of DNA per sample.

Nanopore sequencing was then conducted on ONT’s MinION Mk1B platform using two FLO-MIN106 R9.4.1 flow cells (IDs: FAO38571 and FAO29050), in accordance with the manufacturer’s protocols. Each flow cell was primed and loaded with the prepared libraries, and 48-hour sequencing runs were initiated through MinKNOW software v24.02.8, with MinKNOW’s real-time basecalling option disabled.

### Bioinformatics analyses

After sequencing, raw .fast5 output files were converted to .pod5 files using the Pod5 File Format v0.1.21 tool (Oxford Nanopore Technologies) to optimize overall efficiency. Basecalling, adapter trimming, and quality filtering (Q-score ≥ 9) were carried out with Dorado v0.9.0 (Oxford Nanopore Technologies) using the high-accuracy (HAC) v3.3 model. The resulting quality-filtered reads were then demultiplexed into per-barcode .bam files using the dorado “demux” command. Following demultiplexing, primer removal was performed using Cutadapt v5.0 (quality cutoff: 20) (Martin 2011), and the resulting reads were filtered by length (≥ 400 bp) using Chopper v0.9.1 (https://github.com/wdecoster/chopper; De Coster and Rademakers 2023). Read counts per treatment at each filtering stage were tabulated and visualized in R v4.4.1 (R Core Team 2024) using the tidyverse package (Wickham et al. 2019).

The final filtered/trimmed reads with removed primers were then error-corrected with Canu v2.2 (https://github.com/marbl/canu). Only its error-correction module was employed here to mitigate the moderate per-base accuracy of the ONT R9.4.1 platform prior to clustering. Since Canu-based correction builds consensus sequences, it can sometimes collapse closely related variants when working out with nanopore sequences (Koren et al. 2017); however, as our aim is to compare relative fungal community profiles among sample types rather than to delimit novel taxa or intraspecific variation, this limitation, which is expected to affect all libraries uniformly, has minimal impact on our between-sample comparisons.

Amplicon sequence variants (ASVs) were subsequently generated from the Canu-corrected reads in VSEARCH v2.17.1 (Rognes et al. 2016), using the following steps: dereplication (full length), denoising (cluster_unoise), chimera removal (uchime_denovo), and ASV clustering. Taxonomic assignment of our operational units (ASVs) was carried out using Kraken2 (Wood et al. 2019), which employs a *k*-mer-based lowest common ancestor (LCA) approach. This approach works by splitting the sequences into short genomic substrings (*k*-mers) and comparing those against a specific database, matching the ASVs to their closest available relatives. For our purposes, we compiled a total of 56,186 translation elongation factor 1-alpha sequences (TEF1-α), filtered to Kingdom *Fungi*, from NCBI/GenBank’s non-redundant nucleotide (nt) database. Kraken2 reports for individual samples were then merged by treatment group using the “combine_kreports.py” script from KrakenTools (Lu et al. 2022).

For fungal microbiome visualization, merged reports were analyzed with Pavian (Breitwieser and Salzberg 2020) and converted to Krona-compatible format via the “kreport2krona.py” script (KrakenTools) on the Galaxy platform (The Galaxy Community, 2024). Pavian was used to generate Sankey diagrams at a genus-level, and KronaTools (Ondov et al. 2011) was used to generate interactive hierarchical visualizations. Stacked bar charts depicting fungal community composition at class, order, family, and genus levels were generated using a custom R script employing the tidyverse and patchwork packages (Pedersen 2025).

## Results

### Species inventory and composition

**Figure 3.**
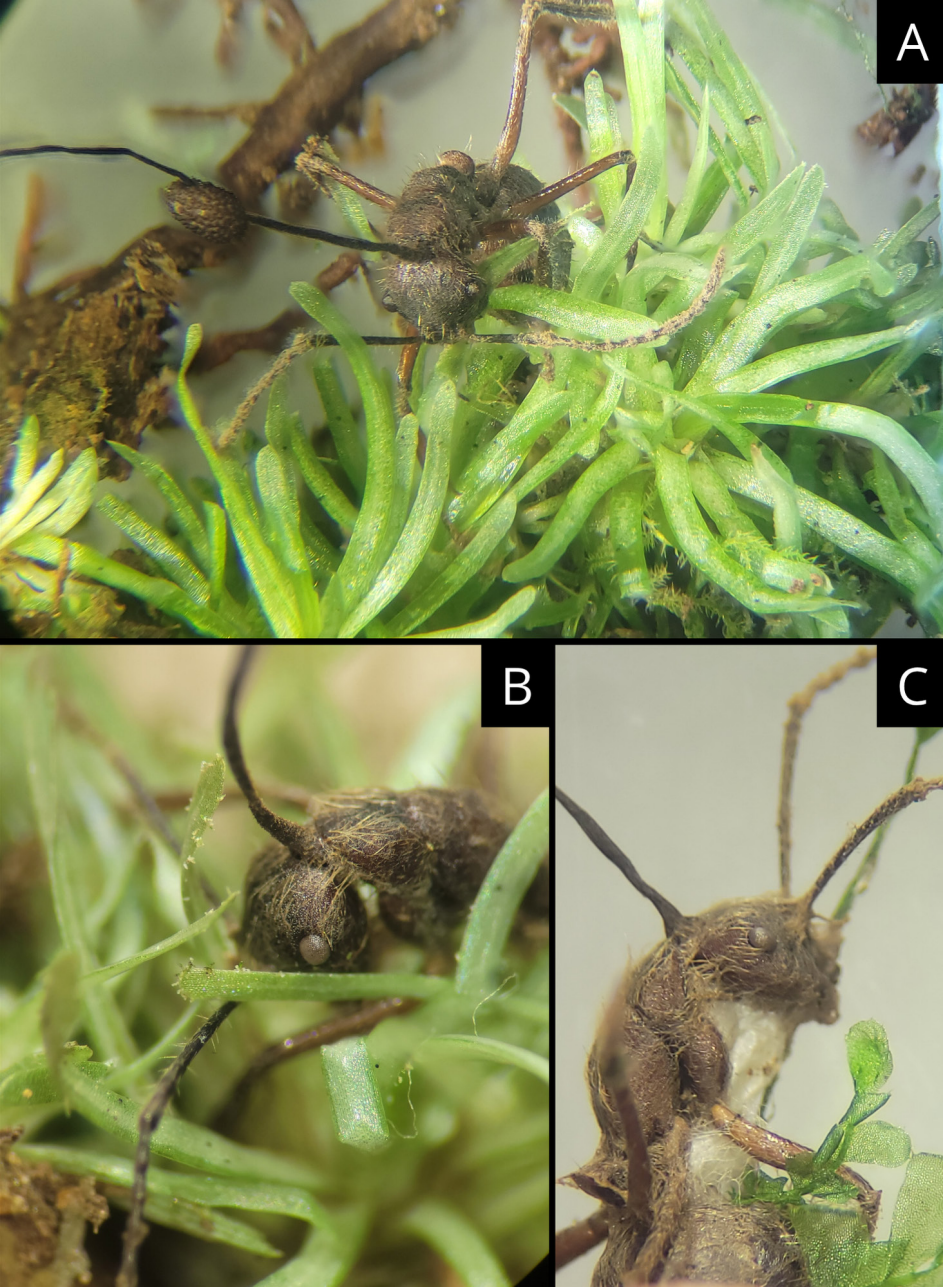
*Camponotus nidulans* ants infected by *O. camponoti-nidulantis* found among bryophytes at the Adolpho Ducke Forest Reserve. **A** Specimen “TAJ59” found on *Octoblepharum* moss; **B** Specimen “TAJ63” also found on *Octoblepharum* moss; **C** Specimen “TAJ60” found biting and grasping an unidentified *Lejeuneaceae* liverwort.

**Figure 4.**
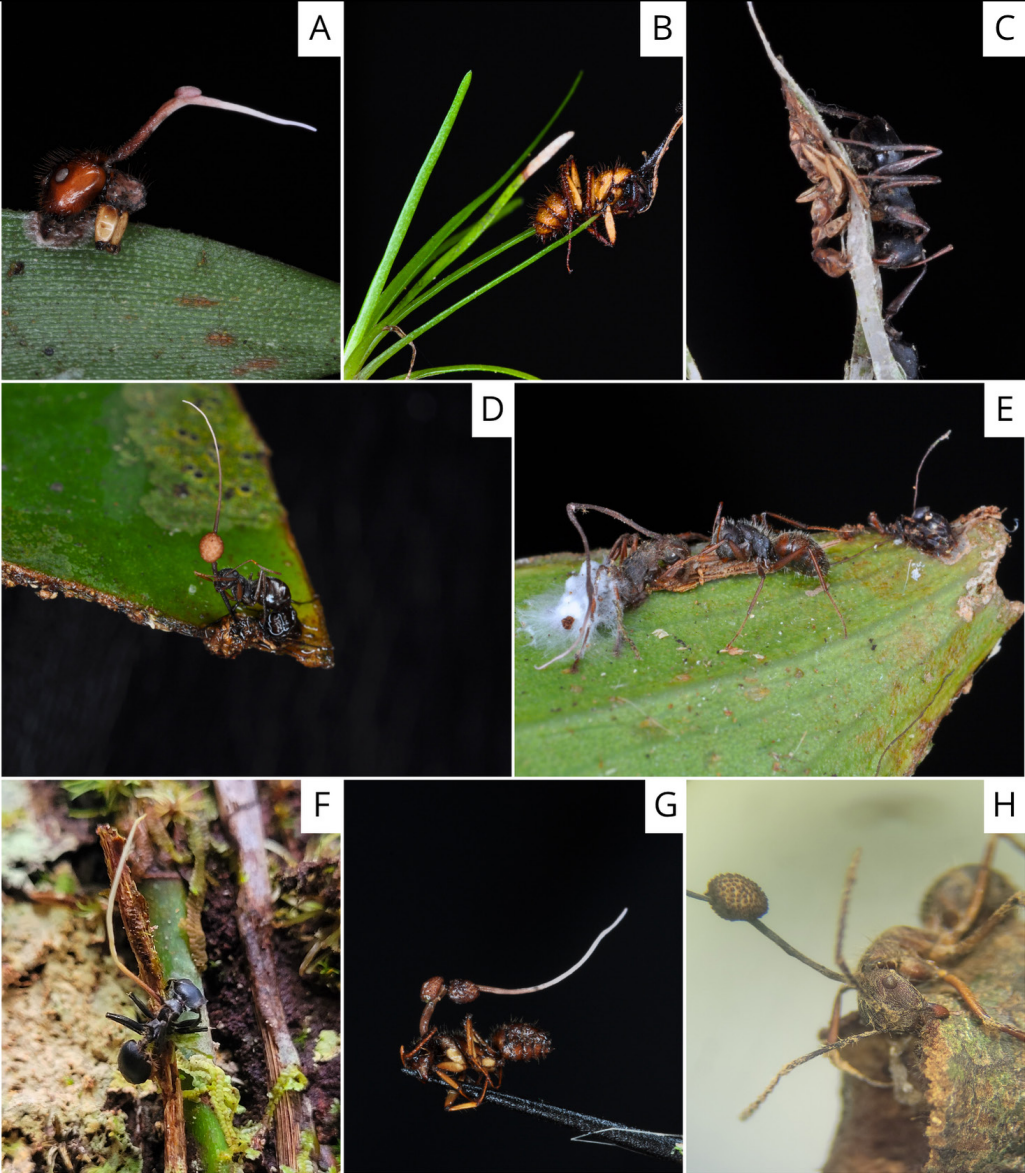
Diversity of biting locations for *Ophiocordyceps*-infected ants. **A***Camponotus* sp. biting a monocot leaf; **B***Camponotus atriceps* infected by *O. camponoti-atricipis* biting a bromeliad; **C** Two different *Camponotus* spp. ants found grasping a leaf; **D***Camponotus* sp. biting another stroma/fruiting body; **E** Mini graveyard containing multiple dead *Camponotus* sp. ants; **F***Cephalotes atratus* embracing an epiphyte; **G***Camponotus* sp. biting into an *Astrocaryum* thorn; **H***Camponotus nidulans* biting onto tree bark.

During field expeditions at the Adolpho Ducke Forest Reserve, we documented a total of 19 *Ophiocordyceps*-infected insects on bryophytes (Table 1). The most dominant group (16/19) comprised the “zombie-ant fungi”, notably *Ophiocordyceps camponoti-nidulantis* (Fig. 3A–C), *O. camponoti-renggeri* and *O. kniphofioides* infecting, respectively, *Camponotus nidulans, C. renggeri* and *Cephalotes atratus*. Among the ants recorded, *Camponotus* was the most frequent (n = 8), followed by *Neoponera* (n = 6) and *Cephalotes* (n = 2) (Table 1). Beyond these bryophyte-associated cadavers, infected ants throughout the reserve were also observed biting a diversity of other substrates (Fig. 4), illustrating the broader range of death-site choices within this system.

**Figure 5.**
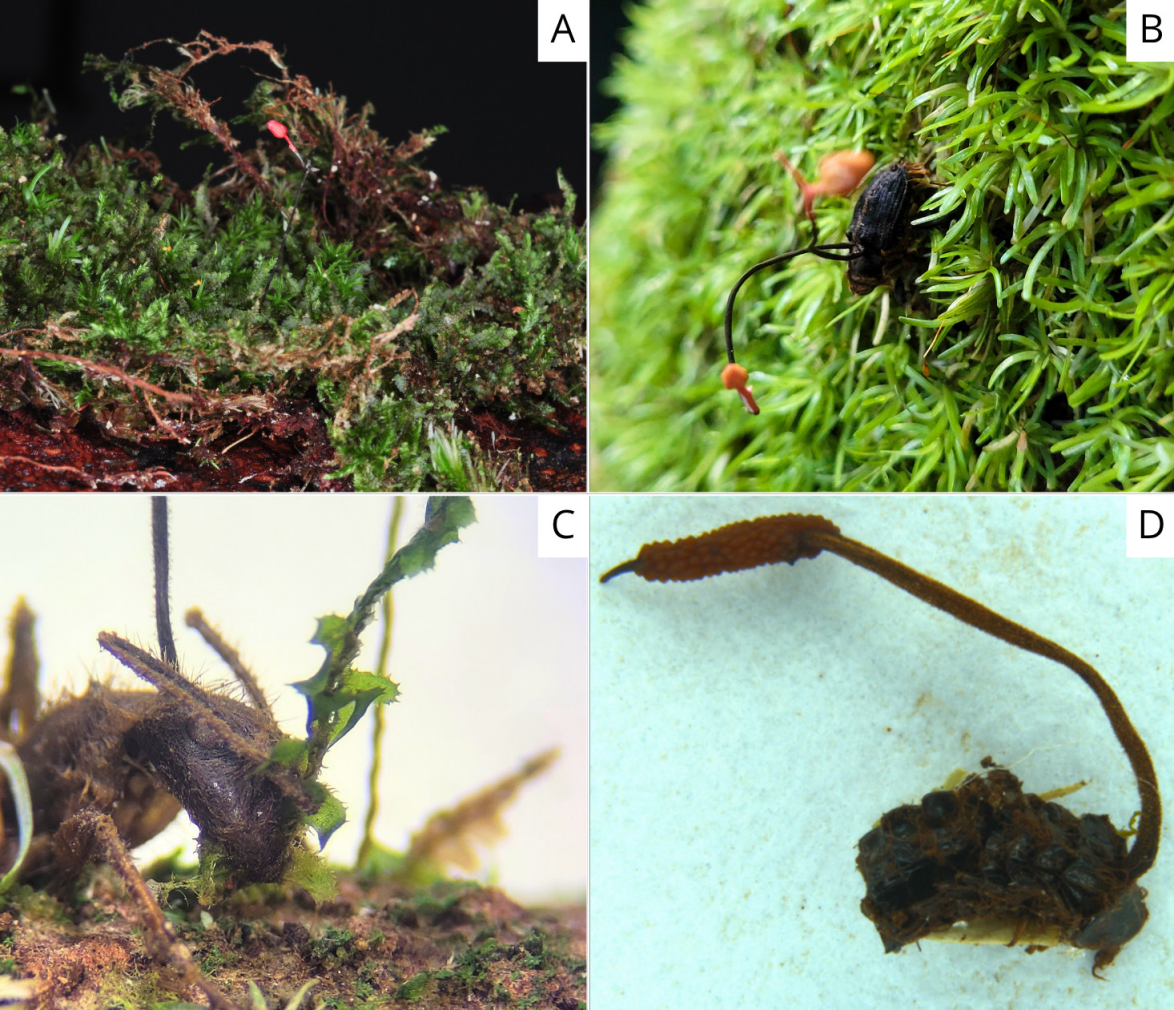
*Ophiocordyceps*-infected insects found attached to bryophytes. **A***Neoponera* ant infected by *O. evansii* (TAJ101), buried into a moss carpet; **B***Piazurus melanostictus* weevil infected by *O. curculionum* (TAJ30) found grasping an *Octoblepharum* moss carpet; **C***Camponotus renggeri* ant infected by *O. camponoti-renggeri* (TAJ32) biting into a *Plagiochila* liverwort while gripping an *Octoblepharum* moss with its tarsal claws; **D***Lampyridae* larva infected by an undescribed species within *Ophiocordyceps kniphofioides* s.l. (TAJ31), grasping an *Octoblepharum* moss.

Other insect groups were also found infected by *Ophiocordyceps*, including a *Piazurus* weevil (n = 1, *Coleoptera*: *Curculionidae*) (Fig. 5B) infected with *O. curculionum*, a *Pintalia* planthopper (n = 1, *Hemiptera*: *Cixiidae*) with *Ophiocordyceps* sp., and a firefly larva (n = 1, *Coleoptera*: *Lampyridae*, Fig. 5D) infected with an undescribed species within *O. kniphofioides* s.l. Most of the infected insects were found attached to *Octoblepharum* mosses (11/19), with one biting into a *Plagiochila* liverwort while also grasping an *Octoblepharum* moss (Fig. 5C); also, a small number of specimens were found embedded within bryophyte carpets containing multiple mosses and liverworts, such as *Ceratolejeunea* (Fig. 5A), where the dense growth made any biting point or attachment difficult to resolve.

### DNA metabarcoding of bryophyte-associated fungal communities

We sequenced 11 of the 19 sample sets (Table 1, bold), each comprising the EPF, INT and EXT categories, together with CONTROL per four out of five trails that contained these sequenced sets (HA, HB, B and AC), and each CONTROL in triplicate. The fifth trail (MET) was not included, as none of its specimens were sequenced. In total, DNA metabarcoding of the bryophyte-associated fungal communities yielded 45 libraries (EPF = 11, INT = 11, EXT = 11; and CONTROL = 12, i.e., four trails in triplicate). Prior to downstream processing, we excluded low-quality or contaminated samples, and *Ophiocordyceps* specimens not parasitizing ants, to standardize sampling and ensure data quality.

**Figure 6.**
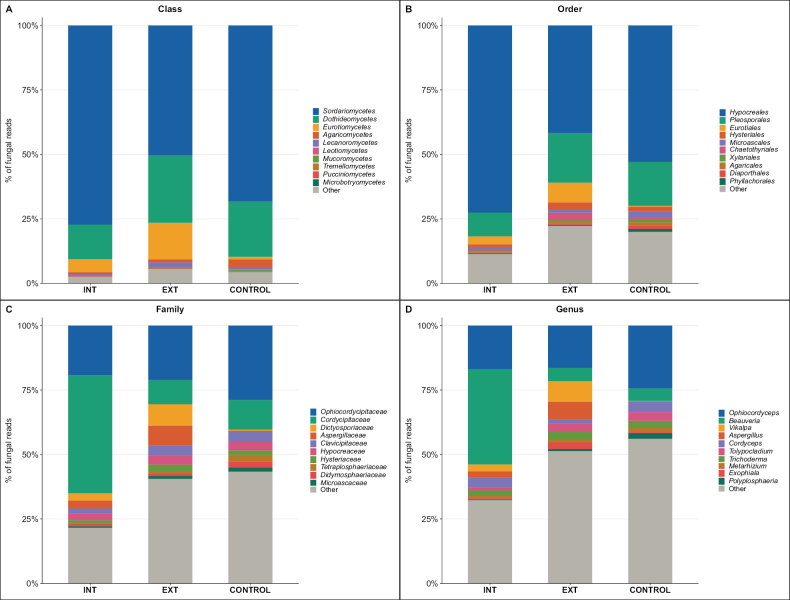
Fungal community composition across bryophyte sample types at class (**A**), order (**B**), family (**C**), and genus (**D**) levels. Bars represent the relative proportion of fungal taxa as a percentage of total fungal reads for each treatment. The top 10 taxa by mean relative proportion are shown individually; remaining taxa are collapsed into “Other.”

**Figure 7.**
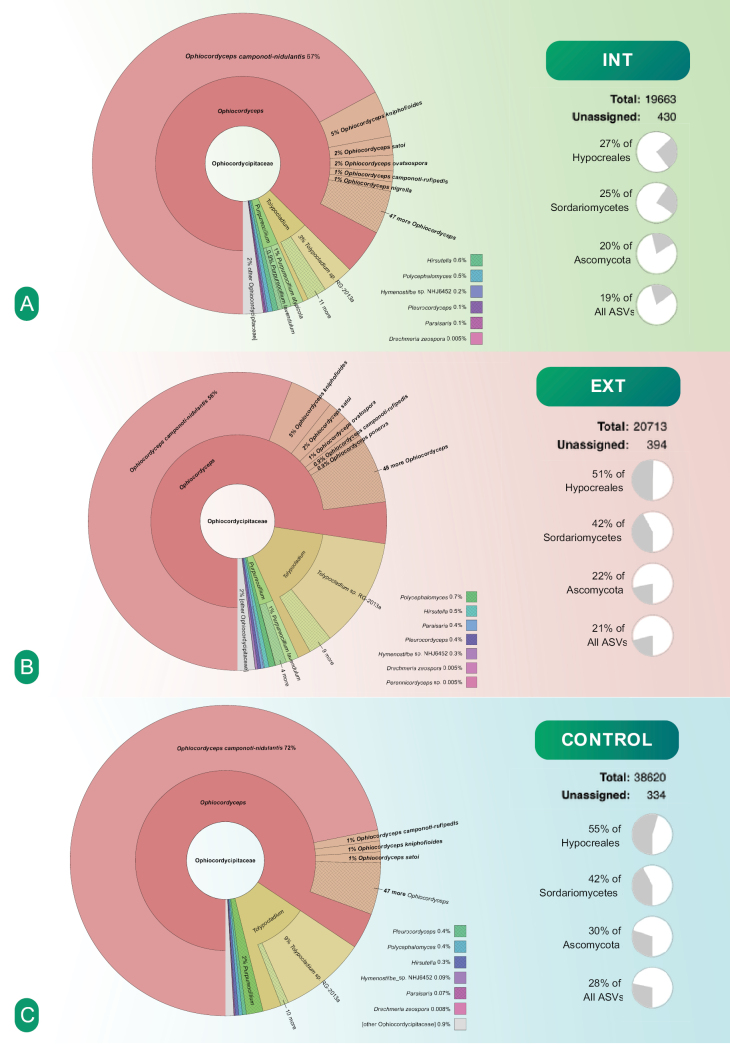
Fungal microbiome Krona plots showing the combined taxonomic assignment for *Ophiocordycipitaceae*ASVs in each treatment: INT (**A**), EXT (**B**), and CONTROL (**C**), highlighting the higher count of *Ophiocordyceps* reads compared to other confamilial genera. At the right corner, pie charts represent the relative proportion of *Ophiocordycipitaceae* reads within different taxonomic levels for INT, EXT and CONTROL.

**Figure 8.**
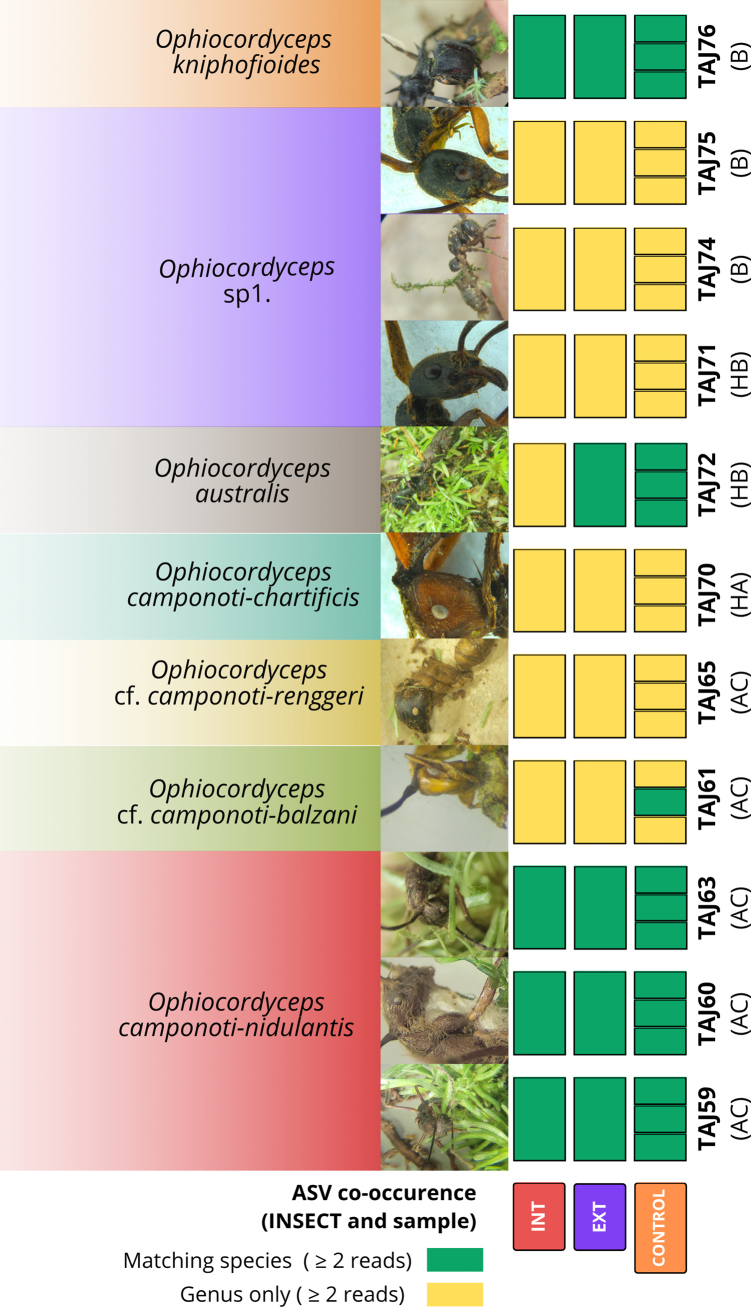
*Ophiocordyceps* lineages detected across bryophyte sample types (INT, EXT, CONTROL) and their respective EPF sample. Boxes marked in green indicate assignment to the same species in both the EPF specimen and the bryophyte sample at ≥ 2 reads; Boxes marked in yellow indicate shared assignments at the genus level (≥ 2 reads) without species-level concordance.

As for our sequenced EPF libraries (*Ophiocordyceps* sampled from the infected hosts), it is worth mentioning that they were excluded from the community-composition comparisons (Figs 6, 7), which focus on the bryophyte-associated mycobiome (INT, EXT and CONTROL). Instead, those libraries serve as a reference group, allowing us to assess whether the same *Ophiocordyceps* lineages detected on the infected insects were also recovered from their corresponding bryophyte samples (Fig. 8).

Following quality filtering, the sequenced libraries yielded a total of 4,289,680 reads (Suppl. material 1: fig. S1). Subsequent length filtering retained 1,058,014 reads (Suppl. material 1: fig. S2), and from this dataset, 490,615 reads were successfully assigned to 2,277 fungal taxa, encompassing four phyla, 29 classes, 324 families, and 886 genera, using our fungal TEF1-α reference database (Fig. 6).

Fungal community profiling revealed significant and consistent presence of entomopathogenic fungi across all bryophyte samples (INT/EXT/CONTROL), including *Ophiocordyceps*, other *Ophiocordycipitaceae* taxa, and insect-associated taxa from other hypocrealean families, such as *Cordycipitaceae* and *Clavicipitaceae* (Figs 6, 7, Suppl. material 1: fig. S3). Of the 490,615 total fungal reads, 62,765 (12.8%) were assigned to 65 *Ophiocordyceps* lineages, emphasizing the genus as a major component of the bryophyte-associated fungal community. Within this group, *O. camponoti-nidulantis* was overwhelmingly dominant, accounting for 52,519 reads (83.7% of all *Ophiocordyceps* reads), consistent with its high incidence in field collections (Table 1), and *O. kniphofioides* ranked second with 2,386 reads (3.8%).

The same *Ophiocordyceps* lineages detected in infected insects (EPF) were also recovered from their corresponding bryophyte samples (INT/EXT/CONTROL) at a species level based on taxonomic assignment, supporting the co-occurrence of these taxa across substrates (Fig. 8, Suppl. material 1: table SS1). In some cases (TAJ70/71/74/75), species-level assignment was not possible due to the absence of reference sequences in GenBank for those lineages; however, all bryophyte samples yielded *Ophiocordyceps* matches at the genus level.

## Discussion

Fungal pathogens, such as those that infect arthropods, frequently expand or shift their host range over evolutionary time as a strategy to reduce fitness costs associated with host scarcity and environmental stress (Li et al. 2024b). This host plasticity, whether through host jumps or gradual range expansions, is sustained by a conserved “arsenal” of effectors: secreted proteins and small molecules that enable colonization of diverse hosts across biotrophic and necrotrophic lineages (Vega et al. 2009; Chaverri and Samuels 2013; Thines 2019).

Such effector-driven flexibility can manifest across different lineages within the fungal kingdom: for instance, in *Ascomycota*, certain pleosporalean dark septate endophytes (DSE) such as *Polydomus karssenii* (*Phaeosphaeriaceae*), and *Laburnicola nematophila* (*Didymosphaeriaceae*) which are able to colonize plant roots, can also parasitize cyst nematode eggs (Knapp et al. 2022; Ashraf et al. 2023). In *Hypocreales*, this inter-kingdom versatility can be exemplified by *Clonostachys epichloë* (*Bionectriaceae*), a known mycoparasite of *Epichloë (Clavicipitaceae)* which can also function as an endophyte in *Epichloë*-infected grasses as well as being an entomopathogen of *Botanophila* flies in a tripartite interaction (Górzyńska et al. 2023; Górzyńska 2025).

A further example of hypocrealean fungi displaying this versatility is *Cordyceps cateniannulata* (*Cordycipitaceae*), an entomopathogenic fungus (EPF) shown to also function as an endophyte of *Coffea arabica* and as a mycoparasite of the coffee leaf rust *Hemileia vastatrix* (Pereira et al. 2024). Most relevant to the present study, this versatility is well documented for certain hypocrealean EPF lineages, termed endophytic insect-pathogenic fungi (EIPF), whose adaptive nutritional modes include a latent endophytic phase in which they concomitantly exploit both plants and arthropods (Branine et al. 2019; Wang et al. 2020). Although such secondary ecological roles have been widely characterized in generalist EPFs such as *Beauveria* and *Metarhizium* (Gange et al. 2019; Stone and Bidochka 2020), they remain unexplored in specialist EPFs which exhibit host behavior manipulation as most noted in *Ophiocordyceps*.

Historically classified as a strict obligate insect pathogen with most species showing narrow host specificity, *Ophiocordyceps* is now further recognized as an EIPF principally due to studies conducted across disparate ecosystems demonstrating its presence in both bryophytes and vascular plants (Davey et al. 2014; Cao et al. 2020; Wang et al. 2020; Matsuoka et al. 2021; Fonseca et al. 2022). Genomic studies have also identified, in certain ant-infecting lineages of this genus (i.e., *O. australis* and *O. polyrhachis-furcata*), effector genes related to plant colonization, demonstrating the broad genetic toolkit that facilitates such ecological plasticity and diversity of life-styles, relating to some basal, mostly plant-associated lineages in *Hypocreales* (Wichadakul et al. 2015; de Menezes et al. 2023).

Our data provide, for the first time, evidence consistent with an endophytic stage in the “zombie-ant fungi” life cycle, particularly highlighting the importance of bryophytes in this system. Furthermore, our results substantially expand scientific knowledge regarding the diversity harbored in endophytic fungal communities of Amazonian bryophytes. While fungal microbiomes have been characterized for Amazonian angiosperms (Vaz et al. 2018; Fonseca et al. 2022), bryophytes in polar (Hirose et al. 2016), temperate (Davey et al. 2013a, 2013b) as well as subtropical zones (Cao et al. 2020), the mycobiome inhabiting Amazonian bryophytes remains heretofore underexplored (Chen and Nelson 2022).

We detected multiple *Ophiocordyceps* species, including known strict host-specific species of behavior manipulating fungi (*O. camponoti-nidulantis* and *O. kniphofioides*) consistently across all bryophyte samples, including the EXT and CONTROL samples (see Fig. 1). Those findings indicate that myrmecophilous *Ophiocordyceps* is spatially widespread among Amazonian bryophytes as endophytes. This recurrent presence, coupled with co-detection of identical/closely related ASVs within bryophytes and associated infected insects, demonstrates the endophyte-entomopathogen lifestyle in manipulative “zombie” *Ophiocordyceps* species, suggesting that such a dual lifestyle is broader across entomopathogenic fungi than currently assumed. Our findings reinforce earlier studies demonstrating *O. sinensis* as both entomopathogenic and predominant endophytic component of several plant families consumed by its caterpillar host (Zhong et al. 2014; Wang et al. 2020; Chen et al. 2024).

Although TEF1-α is a comparatively well represented barcode for *Ophiocordyceps*, its coverage across other fungal groups remains far sparser than ITS databases (e.g. UNITE or GenBank), with many tropical and/or undescribed lineages unrepresented (Meyer et al. 2019). As a result, in several of our bryophyte samples the ASVs matched their respective EPF libraries only at the genus level. We attribute this to the interaction between reference gaps and the Kraken2 LCA approach. Since this software assigns each sequence to the lowest taxonomic node consistent with its k-mers (Wood et al. 2019), reads from species lacking a conspecific reference are placed on a higher node, typically the genus, or on their nearest represented relative. These issues, however, derive more from current knowledge gaps regarding the diversity of this genus rather than a limitation of our approach, and are something that will likely be mitigated in future studies as more sequences become available for this barcode. Hence, expanding the curated TEF1-α reference database for *Ophiocordyceps* and other fungal genera should therefore remain a priority in future works following a similar approach.

Beyond the taxonomic specificity, the biology of some mosses, specifically regarding its anatomical features, may contribute to the presence of *Ophiocordyceps* in higher proportion in our samples. Most of the sequenced sample sets (6 of 11) were associated with *Octoblepharum*, a moss genus whose leaves consist of dead, water-storing cells known as hyalocysts, or leucocysts (Gradstein et al. 2001). Because such structures hypothetically trap environmental spores and shelter them from surface sterilization, the detection might in part reflect retained ascospores rather than endophytic colonization. However, these cells occur in only one of our three sampled bryophyte groups, yet *Ophiocordyceps* was likewise recovered from groups that lack them, namely *Lejeuneaceae* and *Leucomium*, which together account for five of 11 (nearly half) of the sequenced sets. We therefore retain “endophytic” in its operational EIPF sense (Moonjely et al. 2016; Branine et al. 2019; Wang et al. 2020), while recognizing that future studies should consider including techniques that localize and confirm active colonization inside any tissue of the plants, such as histological staining of internal tissue or fluorescence *in situ* hybridization, for instance. Nonetheless, our findings regarding the detection of *Ophiocordyceps* within bryophytes are worth further exploration within an ecological and evolutionary context.

This non-random association among *Ophiocordyceps* and plant substrates, herein demonstrated in a bryophyte-associated stage, may reflect the genus’ evolutionary history over the past 100 million years (Sung et al. 2007a, 2008; Zhuang et al. 2025). Endophytism and plant associations are phylogenetically disparate across *Hypocreales*, as its most recent common ancestor was likely a plant-associated lineage with insect pathogenicity possibly emerging secondarily (Sung et al. 2007a, 2007b; Chaverri and Samuels 2013; Thines 2019). Such endophytic capacity, however, is dynamic as lifestyle transitions in hypocrealean fungi have been demonstrably shaped by introgression and gene-family expansion/contraction (Zhang et al. 2018). For example, a latent plant-associated life mode could be facultatively re-expressed across lineages, facilitating host-range expansion and blurring the distinction between a true host shift and the re-emergence of an ancestral plant-associated state.

### The evolution of substrate fidelity in *Ophiocordyceps*

Over their shared evolutionary history, *Ophiocordyceps* and their arthropod hosts have engaged in coevolutionary dynamics such as directional selection, in which each partner responds to and develops counteradaptations against the other (Buckingham and Ashby 2022). As a result, *Ophiocordyceps* seemingly evolved a suite of fitness-maximizing traits that increase its virulence, infectivity, while also manipulating the host behavior to optimize spore dispersal. Such traits which underly behavioral manipulation are classically interpreted as the “extended phenotype” (Hughes et al. 2011; Dawkins 2016; Andriolli et al. 2024); in quantitative-genetic terms, the same phenomenon may be formalized as an interspecific indirect genetic effect (IIGE), whereby variation in the parasite’s genotype alters the phenotype of its host and the resulting selection feeds back on both partners (de Lisle et al. 2022).

To understand how substrate fidelity related to “death grips” may confer fitness to the EPF, a clearer understanding of its evolutionary history is necessary. Ancestral beetle-infecting lineages in *Ophiocordyceps* preceding ant-infecting *O. unilateralis* s.l. were subjected to strong selective pressure during this host-jump, culminating in the emergence of a sophisticated behavioral manipulation framework needed to overcome the social immunity of ant colonies (Araújo and Hughes 2019). Among such changes, ancestral *O. unilateralis* s.l. lineages induced leaf-biting behavior in ants inhabiting evergreen forests, a trait that posteriorly underwent convergent evolution to twig-biting, likely an adaptive response to changing forest microhabitats in which twigs provide greater temporal stability for fungal development and spore release during seasonal shifts (Loreto et al. 2018).

Substrate specificity may reflect how host-parasite coevolutionary dynamics intersect with environmental conditions and co-occurring organisms in a multitrophic setting, extending well beyond the fungus and its ant host. The high incidence of certain ant-infecting *Ophiocordyceps* in mosses such as *Octoblepharum* (Araújo et al. 2018), bromeliads such as *Tillandsia* (Will et al. 2023a), and understory palms such as *Attalea* and *Euterpe* (Cardoso Neto et al. 2019; Andriolli et al. 2025) are the most common examples in (sub) tropical forests. Nevertheless, despite considerable progress in disentangling the mechanistic, molecular, biochemical, and ecological underpinnings of host manipulation by *Ophiocordyceps* (de Bekker et al. 2017, 2021; Will et al. 2020, 2023b), the importance of substrate fidelity remains an intriguing mystery ripe for further investigation.

Our data address critical knowledge gaps regarding an endophytic phase in bryophytes by ant-infecting *Ophiocordyceps* lineages and refine our understanding of niche selection as exemplified by biting behavior in specialist EPF lineages within this genus. Although previous studies have framed plants as infection vectors for larval hosts (Wang et al. 2020; Chen et al. 2024), further research should examine niche expansion dynamics, addressing questions such as: (1) Does endophytism function to enhance environmental persistence during host/nutritional/environmental scarcity?; (2) Are fungal effectors and/or volatile organic compounds (VOCs) drivers of the plant host specificity?; and finally (3) Do plants function as ‘mating arenas’ enabling genetic exchange among spatially separated strains in heterothallic EIPF systems? Whether these questions will elucidate how coevolutionary dynamics shape cross-kingdom interactions and transform plant substrates into components within this multi-layered interaction framework remains to be tested.

## Conclusions

Our study provides evidence consistent with an endophytic lifestyle of manipulative myrmecophilous *Ophiocordyceps* on Amazonian bryophytes, challenging the historical designation as obligate strict insect pathogens. Using metabarcoding of stratified bryophyte samples, we detected several host-specific *Ophiocordyceps* lineages. This aligns with findings from temperate and subtropical regions on other continents and, to our knowledge, is the first test of the presence or absence of these manipulative fungi in neotropical bryophytes.

Additionally, the co-detection of identical or closely related ASVs in bryophytes and the associated infected insects indicates that the endophyte-entomopathogen lifestyle in this genus is not random, but likely reflects an evolutionarily conserved capacity for plant association, as observed in other hypocrealean fungi, expressed alongside the specialized entomopathogenic stage and plausibly contributing to the fungus’s persistence and reproduction. Elucidating how entomopathogenic fungal lineages establish these multi-layered interactions may therefore prove critical to understanding cross-kingdom and coevolutionary processes in Amazonian ecosystems.

## Additional information

### Artificial Intelligence (AI) use

Generative Artificial Intelligence tools (Claude Sonnet 4.5/Opus 4.8, Anthropic; Gemini Pro 3.1, Google) were used by the authors solely to assist with linguistic refinement and structural editing to improve overall reading clarity, as well as to support data visualization coding and AI-assisted image generation. No content was generated by AI as part of the formal research design, data collection, or interpretation, and no AI tool is listed as an author.

### Adherence to national and international regulations

Field sampling was conducted with the necessary permits to access Adolpho Ducke Forest Reserve given by INPA and complied with applicable institutional and national guidelines for biodiversity research in Brazil, respecting the Convention on Biological Diversity (Rio Convention). No human participants or vertebrate animals were involved in this study. SISBIO/ICMBio Authorizations 87724-1 and 87721-2.

### Author ORCIDs

T. Alves-Júnior https://orcid.org/0000-0003-4243-9794

J.P.M. de Araújo https://orcid.org/0000-0003-2529-7691

A. Góes-Neto https://orcid.org/0000-0002-7692-6243

T. Mendes-Pereira https://orcid.org/0000-0002-3707-9790

C.E. Zartman https://orcid.org/0000-0001-8481-9782

### Data availability

All experimental data pertinent to this manuscript are available for review within the manuscript. Additionally, all Kraken2 taxonomic assignments (both individual and grouped), read counts, original Krona plots and Pavian graphs (in .html), as well as the processed nanopore reads in .fastq are available at Zenodo (https://doi.org/10.5281/zenodo.18965795).

## Supplementary materials

Supplementary material 1 Supplementary images and tables Data type docx https://binary.pensoft.net/file/1715841This dataset is made available under the Open Database License (http://opendatacommons.org/licenses/odbl/1.0/). The Open Database License (ODbL) is a license agreement intended to allow users to freely share, modify, and use this Dataset while maintaining this same freedom for others, provided that the original source and author(s) are credited. Tales Alves-Júnior, João Paulo Machado de Araújo, Aristóteles Góes-Neto, Thairine Mendes-Pereira, Charles E. Zartman
