# Supplementary material for: Metabarcoding reveals a hidden endophytic stage of the 'zombie-ant' fungus in Amazonian mosses
Source: IMA Fungus. 2026 Jul 14;17:e196998. doi: 10.3897/imafungus.17.196998 (PMC13389564; doi:10.3897/imafungus.17.196998)
Supplement: Supplementary material 1 — Supplementary images and tables [file imafungus-17-e196998-s001.docx]

**APPENDIX**

**
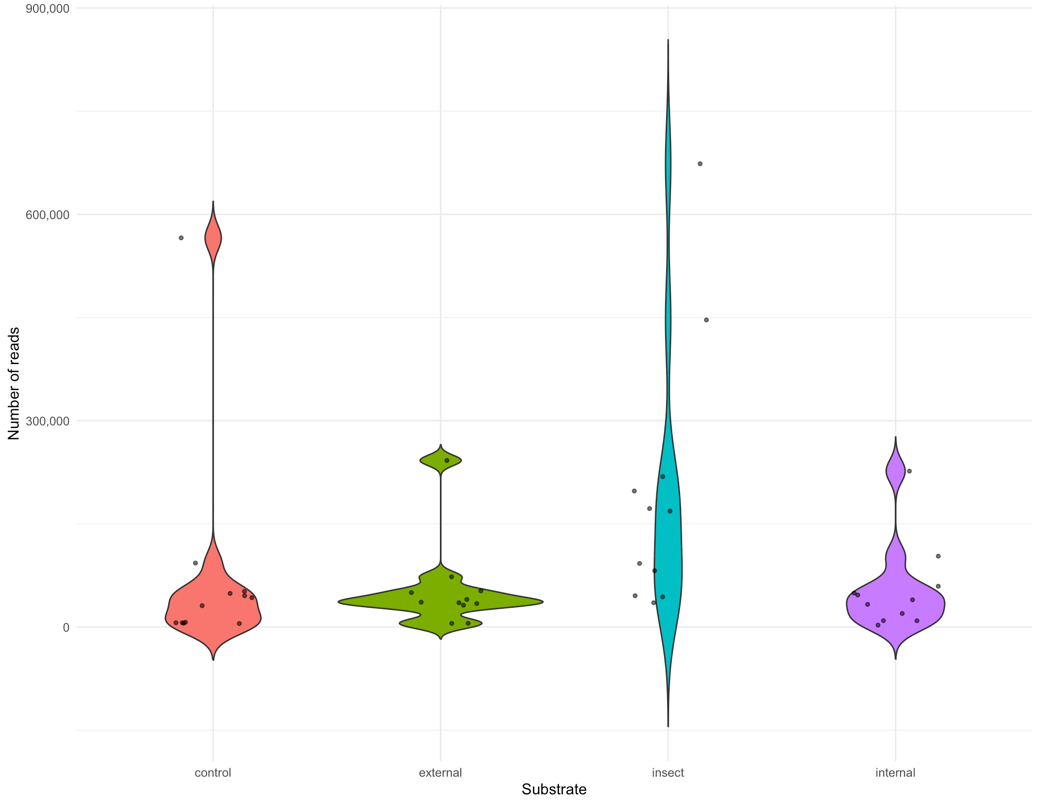
**

**Figure S1**. Resulting reads after being filtered by quality (QC>9), grouped by substrate.


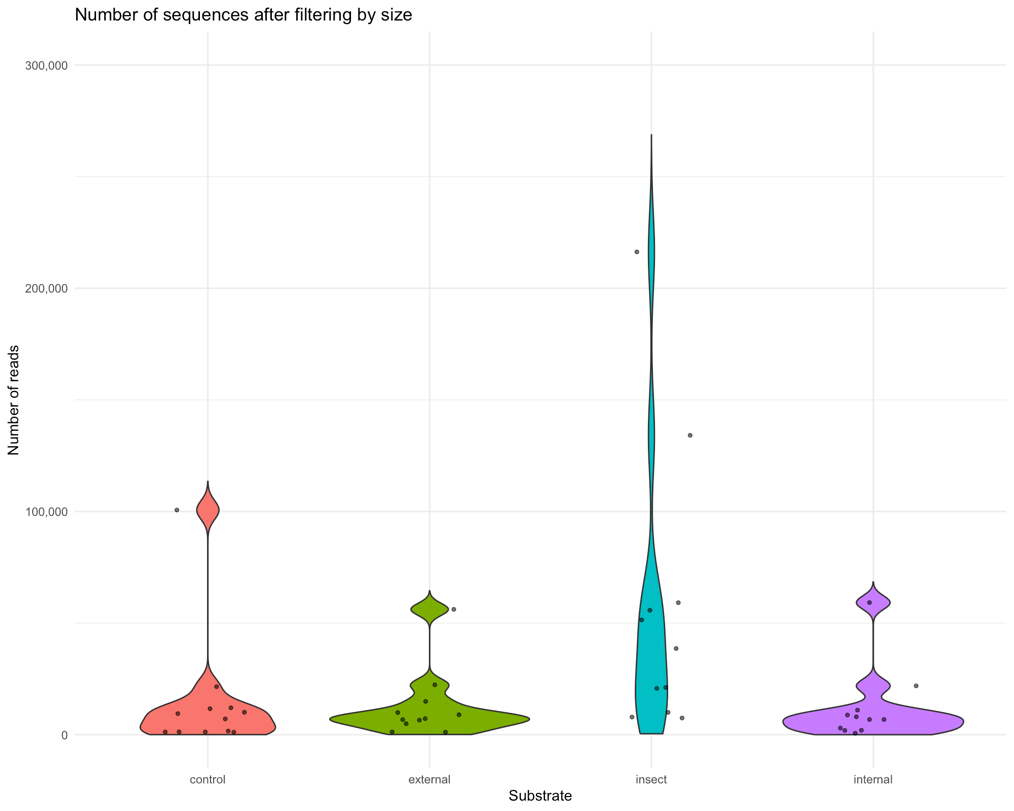


**Figure S2**. Resulting reads after being filtered by quality (QC>9) and length (≥500 bp), grouped by substrate.


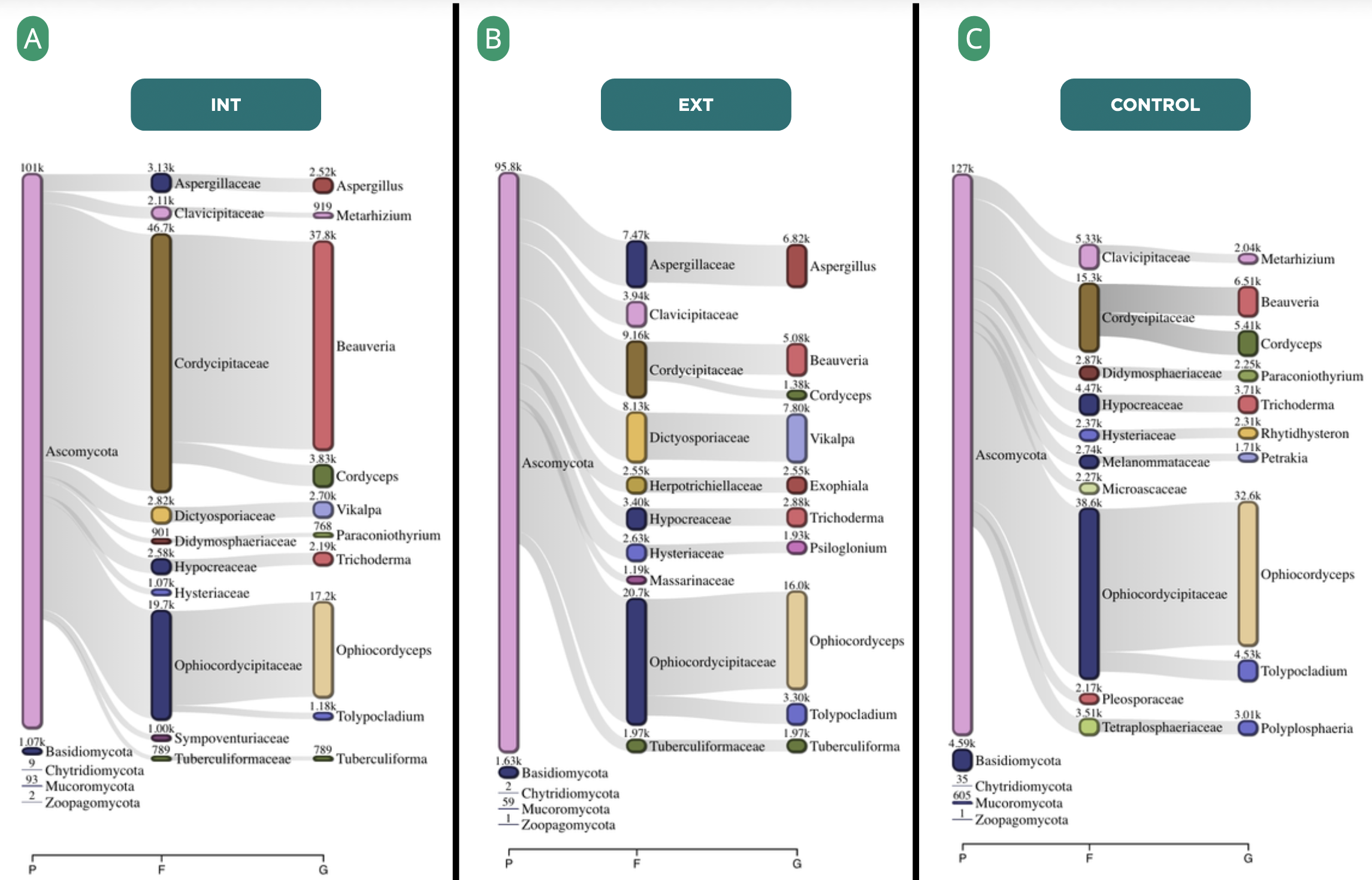


**Figure S3.** Supplementary Figure 1: Pavian-style Sankey plot visualization of taxonomic assignments for INT (A), EXT (B), CONTROL (C), showing number of sequences at Phyla, Family, or Genus’s level.

**Table S1.** Percentage of *Ophiocordyceps* reads detected within bryophytes showing co-occurrence of identical/similar lineages (at species level) between bryophyte samples (INT/EXT/CONTROL) and their respective INSECT sample. Singletons (≤1 read) were considered as 0% within our data. Samples marked with an asterisk means that there’s no reference sample within GenBank for that fungal species. CONTROL reads are respective to the trail that the sample was collected (see Table 1), and the respective fungal species cited.

| ID | *Species (INSECT)* | *INT*  *(% Reads)* | *EXT*  *(% Reads)* | *CONTROL1*  *(% Reads)* | *CONTROL2*  *(% Reads)* | *CONTROL3*  *(% Reads)* |
| --- | --- | --- | --- | --- | --- | --- |
| TAJ59  (AC) | *Ophiocordyceps*  *camponoti-nidulantis* | 1.01% | 0.46% | 42.98% | 54.62% | 63.54% |
| TAJ60  (AC) | *Ophiocordyceps*  *camponoti-nidulantis* | 48.61% | 41.83% | 42.98% | 54.62% | 63.5% |
| TAJ61  (AC) | *Ophiocordyceps*  *cf. camponoti-balzani* | 0%  (genus: 41.29%) | 0%  (genus: 31.98%) | 0%  (genus: 58.84%) | 0%  (genus: 53.56%) | 0.03% |
| TAJ63  (AC) | *Ophiocordyceps*  *camponoti-nidulantis* | 5.15% | 24.19% | 42.98% | 54.62% | 63.54% |
| TAJ65  (AC) | *Ophiocordyceps*  *cf. camponoti-renggeri* | 0%  (genus: 28.07%) | 0%  (genus: 58.17%) | 0%  (genus: 58.84%) | 0%  (genus: 53.56%) | 0%  (genus: 64.47%) |
| TAJ70*  (HA) | *Ophiocordyceps*  *cf. camponoti-chartificis* | 38.98% (genus) | 48.94% (genus) | 68.04% (genus) | 10.8% (genus) | 52.29% (genus) |
| TAJ71*  (HB) | *Ophiocordyceps sp1.* | 83.68% (genus) | 19.4% (genus) | 67.31% (genus) | 23.92% (genus) | 76.23% (genus) |
| TAJ72  (HB) | *Ophiocordyceps australis* | 0%  (genus: 29.53%) | 0.08% | 1.14% | 0.07% | 1.7% |
| TAJ74*  (B) | *Ophiocordyceps sp1.* | 55.45% (genus) | 44.68% (genus) | 66.94% (genus) | 47.92% (genus) | 79.2% (genus) |
| TAJ75*  (B) | *Ophiocordyceps sp1.* | 18.24% (genus) | 21.89% (genus) | 66.94% (genus) | 47.92% (genus) | 79.2% (genus) |
| TAJ76  (B) | *Ophiocordyceps kniphofioides* | 8.86% | 13.1% | 0.11% | 0.06% | 12.21% |
